# Management of chyle leak in right side neck dissection: a rare case and review of literature (a case report)

**DOI:** 10.11604/pamj.2021.40.209.30496

**Published:** 2021-12-07

**Authors:** Rahul Deshmukh, Purva Kulkarni, Umesh Bhutekar, Atul Kala, Shivam Richhariya, Hitesh Tawari

**Affiliations:** 1 Department of Dentistry, Dr. Panjabrao Deshmukh Memorial Medical College, Amravati, India,; 2 Department of Facio-Maxillary Surgery, Sanjay Gandhi Institute of Trauma and Orthopeadics, Bangalore, Karnataka, India,; 3 Department of Oral and Maxillofacial Surgery, Saraswati Dhanwantri Dental College and Hospital, Parbhani, India,; 4 Department of Maxillofacial Surgery, Index Institute of Dental Sciences, Indore, India,; 5 Department of Oral and Maxillofacial Surgery, Ekdant Multi-speciality Dental Clinic, Tilak ward, Deori Kalan, Sagar, Madhya Pradesh, India,; 6 Department of Oral and Maxillofacial Surgery, Maitri Dental College and Research Centre, Durg, Chattishgarh, India

**Keywords:** Chyle leak, thoracic duct, lymphatic, head and neck surgery, case report

## Abstract

Chyle leak is a well-recognized iatrogenic thoracic duct injury but a rare and serious complication of head and neck surgery affecting 1-2.5% of head and neck surgery dissections. It is potentially a life-threatening condition and management may be problematic and prolonged. Here we presented a rare case report of right sided chyle leak with its surgical management and review of literature. A 56-year-old patient with a complain of non-healing ulcer in the right buccal vestibule in the last 1-2 months reported to the outpatient department (OPD). After complete preoperative profile and counseling patient's consent was taken and wide local excision of lesion was done with bite composite resection with right hemimandibulectomy and maxillary alveolectomy till pterygoid plates, with right side selective neck dissection, level I-III followed by reconstruction with right side pectoralis major myofascial flap. Then the patient was on 5 days octreotide therapy. Regular post-operative follow-up was taken and no leak was noted further. In case of a chyle leak early diagnosis and aggressive treatment is essential to avoid local and systemic complications that prolong hospitalization.

## Introduction

Chyle leak is a well-recognized iatrogenic thoracic duct injury but rare and serious complication of head and neck surgery affecting 1-2.5% of head and neck surgery dissections [1]. It is potentially a life-threatening condition and management may be problematic and prolonged. The thoracic duct is the primary structure that returns lymph and chyle from the entire left and right lower half of the body. The variable anatomy of thoracic duct and its fragile composition makes it prone to intra operative damage. The thoracic duct runs from the superior aspect of the cisterna chyli (L2 vertebra), first on the right side, then on the left side (T4 vertebra), eventually curving posteriorly to finish at the left venous angle in the neck. Variations in termination also exist [2-4]. It can end on the left side in the “internal jugular vein (46%), the jugulo-venous angle (32%), the subclavian vein (18%), the external jugular vein, the vertebral vein, the transverse cervical vein, the brachiocephalic vein, the suprascapular vein, the innominate vein, and the right internal jugular vein (4%)” [5]. In each upper body side there are 3 main lymphatic trunks: jugular, subclavian and broncho-mediastinic. On the right, they more frequently end separately, the confluence in a single duct (right lymphatic duct, (RLD)) being less common. The lenght of RLD ranges 10 to 12mm.

Chyle production can be 2-4 L/day, flow of which is dependent on diet, intestinal function, mobility of the patient, respiration and positive intra- abdominal pressure, e.g. coughing. Chyle is composed of proteins, chylomicrons, and electrolytes such as sodium, potassium, chloride and glucose at similar values to those in plasma. Calcium is also present along with WBCs mostly T lymphocytes [6]. The incidence of chyle leak is alarming, as it exposes patients to severe complications, significantly increases their time in hospital and probability of reoperation- in severe cases, it may lead to death due to risk of nutritional and immune compromise, hydro electrolytic disorders, hypoalbuminemia, lymphopenia, surgical wound infection and pleural effusion [7]. Here we presented a rare case report of right sided chyle leak with its surgical management and review of literature.

## Patient and observation

**Patient information:** a 56-year-old patient with a complaint of non healing ulcer in the right buccal vestibule for the past 1-2 months reported to the OPD. Historical examination of patient revealed that there was a change in consistency of saliva from thin to thick with history of exfoliation of tooth along with loss of appetite. The patient was habituated to smoking and chewing tobacco. Family history was positive for oral malignancy.

**Figure 1:**
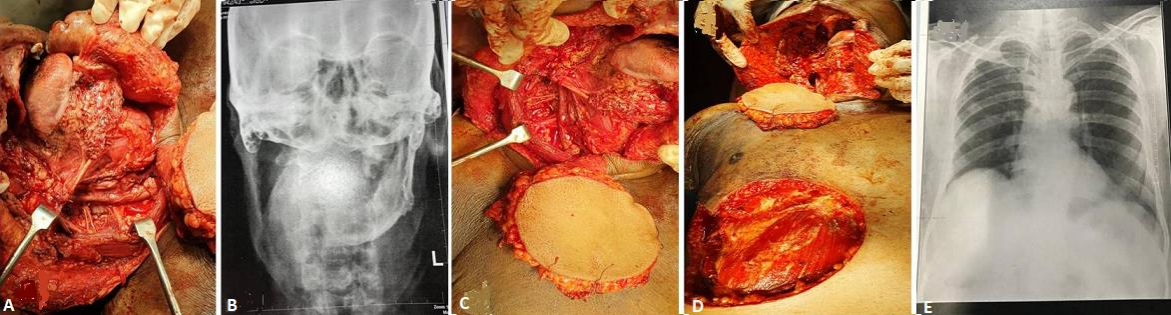
A) intra operative picture showing right bite composite resection involving hemimandible and maxillary alveolectomy till pterygoid plates, with right side selective neck dissection; B) post-operative radiograph of the mandible after bite composite resection (PA view); C) intra operative photograph of pectoralis major myocutaneous (PMM) flap; D) intra operative picture showing no leak of the chyle fluid; E) chest X-ray taken on post-operative day 7 to rule out pleuritis or pleural tear

**Clinical findings**: on thorough examination there was diffuse swelling present on right side of face over lower jaw, mouth opening was restricted and ulceroproliferative growth was seen in right buccal mucosa involving gingivo-buccal sulcus extending to lingual mucosa, edges were everted, lymphadenopathy-submandibular lymph node was palpable and enlarged (0.5 * 1 cm approx), with right side selective neck dissection (Figure 1A and B) level I-III followed by reconstruction with right side pectoralis major myofascial flap (Figure 1C). The intraoperative procedure went uneventful, IJV was preserved and the patient was asked to forcefully breathe out (expiration) against a closed glottis which revealed no incidence of chyle leak intra operatively before closure (Figure 1D). Two drains were secured in neck and chest and patient was shifted in surgical ICU for further monitoring and recording (Figure 1E). The patient was extubated on the first postoperative day and post nothing by mouth (NBM) feeding through nasogastric (NG) ryles tube was started with clear fluids, collection in both the drain was less than 70ml. Day 1: no significant change was there and collection in drain was less 100ml with intermittent column. Day 2: neck flap was healthy and drains had less than 100ml collection. Day 3: mild collection was noticed in chest on the 3^rd^ postoperative day and the chest drain column turned milky/creamy, the output in chest drain increased a bit but it was less than 150ml. Day 4: aspiration was done in the chest and the total chyle collection was around 120ml, the patient was shifted on complete parenteral nutrition and on plain water through NG tube. Complete immobilization was instructed, and sample was sent for further biochemical reporting.

**Diagnostic assessment:** day 5: the biochemical report stated increased number of chylomicrons and triglycerides. Serum electrolyte showed a narrow fall in Na/K levels and total protein. Total leak was still 100-150ml.

**Therapeutic intervention:** day 6: octreotide therapy was started with the dosage being 100μg SC 8 hourly. Aspiration was 240ml from chest. De-epithelization of flap was seen intraorally. Day 7: patient was encouraged to take turmeric water, plain salt and sugar water through NG tube. Medium Chain Fatty Acid (MCFA) along with protein and multivitamin supplements were given. Soakage in neck dressing was seen and total drain collection was 180ml. Day 8: signs of cuticular necrosis (flap necrosis) were seen and detachment from the maxillary tuberosity was noted, though the floor of the mouth was intact. Drain collection was nearly 200ml. Day 9: post octreotide therapy and with proper wound care and aspiration with total parenteral nutrition (TPN) and complete immobilization, chyle was persistent, patients economic, social and physical status was degrading and referral to higher center for re exploration through thoracoscopic ligation was not possible. Negative pressure dressing wasn't done due to economic issues. Re-exploration by surgical method was decided as no conservative management was working and flap was in a compromised state with the chyle leak being 150-200ml (neck and chest drain were removed). Octreotide therapy was discontinued. Day 10: leak was anticipated surgically through existing incision near the junction of sternocleidomastoid muscle (SCM) and internal jugular vein (IJV), pectoralis major flap was dissected from the SCM, SCM was skeletonized to identify the lymphatic duct, patient was kept on positive pressure through bag ventilation by the anesthetist and slight color change was seen, and the suspected leak was ligated and stuffed with 4 sheets of gel foam as a barrier, after counterchecking the same and maintaining proper hemostasis closure was done, drain was secured. Day 11: collection was bloody with intermittent column which was less than 50ml. Day 12: no sign of chyle was anticipated.

## Discussion

All lymphatic fluid produced by the body eventually passes through two channels: the thoracic duct, draining classically into the left subclavian vein, and the right lymphatic duct, draining into the right inominate vein at the junction of the right subclavian and right IJVs. The thoracic duct is the primary structure that returns lymph from the left body and the right body below the diaphragm to the venous circulation. This includes chyle derived from intestinal lacteals [8,9]. The thoracic duct serves a crucial role in the maintenance of fluid balance and return of lymph and chyle to the systemic circulation [10].

Chyle is composed of lymphatic fluid and chylomicrons from the gastrointestinal system. Its lymphatic fluid contains protein, white blood cells, electrolytes, fat-soluble vitamins, trace elements, and glucose absorbed from the interstitial fluid, to be returned to the systemic circulation [11]. Chylomicrons consist of esterified monoglycerides and fatty acids combined with cholesterol and proteins. These are formed from the breakdown products of long-chain fatty acids by bile salts and absorbed into the lymphatic system through special lymphatic vessels in the villous region of the intestines known as lacteals. Conversely, the smaller short and medium-chain fatty acids are more water soluble and are absorbed via the intestinal mucosa directly into the hepatic portal vein, thus by passing the lymphatic system [12]. The majority of chyle leak (CL) transpires with surgery of the left neck; however, up to 25% of CL occur with right neck surgery [13,14]. Although uncommon, CL would surely be encountered in any head and neck surgery practice. Early identification and appropriate management of a CL are imperative for optimal surgical outcome. According to study of Cherian *et al*. [15] the incidence of right sided chyle was found in 24% of the patients leak. It has been evident from the published literature that the incidence of right sided chyle leak is very rare as compared to left side. Our patient presented with a right-sided lymphatic duct injury following a selective neck dissection (level I-IV). His presentation was uncharacteristic as the chyle leak was on the right side of the neck. Chyle leak due to lymph vessel injury during neck surgery has a 2% incidence, and of the 2% only one fourth occur on the right side of the neck [16].

The diagnosis of chyle, chylous ascites, or chylothorax is largely clinical. To confirm the diagnosis, ascitic or pleural fluid is assayed. The presence of chylomicrons and a triglyceride level higher than 110 mg/dL confirm the diagnosis of a chylous leak. The presence of chyle may be confirmed in the laboratory by measuring fat and protein content, pH, and specific gravity. Chyle has a fat content of 0.4-4.0 g/dL, a protein content of approximately 3 g/dL, a pH of greater than 7.5, and a specific gravity of greater than 1.010 g/dL [17]. This rare presentation can contribute to a difficult diagnosis if the provider is not careful. Fortunately, with the visualization of the draining fluid, a formative diagnosis of chyle leak can be easily made by the appearance of a 'milk' quality or white color.

After trying all forms of conservative management with minimal physical activity, dietary management, wound care and 5 days octreotide therapy, surgical re-exploration was considered in the present study. There has been much debate about the exact time and criteria for surgical re exploration. Recent publications on chyle leakage report state that if no improvement is seen within 3 days from the initiation of treatment it is unlikely that there will be a favorable response. Though VATS i.e. Visually Assisted Thoracoscopic Surgery, with thoracic duct ligation have a reported success rate greater than 90% with proximal ligation to the diaphragm [18], but in our case patient was physically and financially compromised and due to lack of availability of VATS as an surgical option, this could not be done, and referral to higher center was not feasible for the patient. The chyle leakage was not more than 250ml, since as all the conservative methods failed and with the higher risk of necrosis of flap and fistula in neck was still present which could have worsened the situation thus surgical re exploration was considered.

Bed rest is recommended, as activity increases chyle output. Suction drains placed in the wound bed at operation allow recognition of chyle leak and also monitoring of drain output. Application of a pressure dressing to create a local tamponade effect is not recommended, as it may be ineffective and may interfere with blood supply to skin flaps. Standard management also includes measurement of fluid input and output and daily urea and electrolytes. Liver function tests including albumin are also indicated to help guide dietary modification and therapy. The dietary management of patients with a persistent chyle leak is important for several reasons. The loss of volume and electrolytes must be replaced, and the nutritional status of the patient must be maintained. It is also possible to use dietary manipulation to attempt to decrease the production of chyle. Most authors recommend an elemental diet with medium-chain triglycerides (MCTs) as an initial dietary response to chyle leakage [19,20]. As detailed previously, MCTs are absorbed directly into the portal venous circulation and as such do not enter the lymphatics, decreasing the output of chyle. Indeed, some authors have reported that this alone may be sufficient to stop chyle leakage. In our case in spite of trying this conservative method of dietary modification and complete immobilization, there was no significant change in the chyle collection.

Treatment with somatostatin, or its synthetic analogue octreotide, is a potential alternative to surgical management of chyle leaks. These drugs have many inhibitory functions on hormones, but their proposed effects on closing chyle leaks derive from their abilities to decrease absorption of triglycerides and inhibit splanchnic circulation and gastrointestinal motility, all factors that affect lymph flow. Octreotide's advantage over somatostatin is that it does not require continuous intravenous infusion, but rather, use of subcutaneous injection can sustain long-lasting effects. Octreotide has emerged as a powerful adjunct in the conservative management of CL and should be a part of the armamentarium of every head and neck surgeon. However, not every CL will respond completely to octreotide therapy alone. Given that octreotide therapy can reduce hospital length of stay, it appears to be cost-effective. In our case octreotide 100μg SC 8 hourly was administered, but we did not observe any significant reduction in chyle collection.

In our case, after trying all conservative methods given in the literature, we did not find any significant change in chyle collection, after this patient was suggested to be on high fatty diet 3 hours before the surgery so as to find the chyle leaking site intraoperatively, after opening the neck the pectoralis major myocutaneous (PMMC) muscle flap was glued with the underlying structures, thus after locating the leak gel foam soaked in thrombin were placed at the leaking site. Drain was secured and closure was done. Regular post-operative follow-up was taken and no leak was noted further.

## Conclusion

Detailed understanding of anatomy of thoracic duct and knowledge of variation helps surgeon to avoid inadvertent injuries. Chylous fistula is a bilateral threat and same care and precaution should be taken on the right as on the left side. In case of a chyle leak early diagnosis and aggressive treatment is essential to avoid local and systemic complications that prolongs hospitalization.
